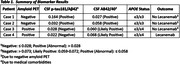# Amyloid Biomarker Discordance in Patients Evaluated for Anti‐Amyloid Therapy

**DOI:** 10.1002/alz.095777

**Published:** 2025-01-09

**Authors:** Vijay K. Ramanan, Jonathan Graff‐Radford, Joshua A Bornhorst, Derek R. Johnson, Petrice M Cogswell, Brian J Burkett, Val J. Lowe, Nikki H. Stricker, Hugo Botha, David T. Jones, Stuart J McCarter, Bryan J. Neth, Clifford R. Jack, David S. Knopman, Ronald C. Petersen, Alicia Algeciras‐Schimnich

**Affiliations:** ^1^ Department of Neurology, Mayo Clinic, Rochester, MN USA; ^2^ Mayo Clinic, Rochester, MN USA; ^3^ Mayo Clinic, Radiology, Rochester, MN USA; ^4^ Department of Radiology, Mayo Clinic, Rochester, MN USA

## Abstract

**Background:**

Evidence for abnormal amyloid‐β (Aβ) plaque accumulation is necessary prior to initiating anti‐amyloid therapy in early symptomatic Alzheimer’s disease (AD). While the clinical trials for lecanemab and related drugs utilized positron emission tomography (PET) to demonstrate brain amyloidosis, current appropriate use recommendations for clinical practice consider PET or cerebrospinal fluid (CSF) biomarkers as satisfactory for this purpose. Here, we present four clinical cases where CSF biomarker results were discordant from amyloid PET, with the potential to result in erroneous treatment targeting.

**Method:**

Patients were seen at a tertiary care subspecialty clinic in consideration for lecanemab therapy. All patients had office examination, neuropsychological assessment, structural magnetic resonance imaging (MRI), amyloid PET (with either ^11^C‐Pittsburgh compound B or ^18^F‐florbetapir), CSF AD biomarkers (Roche Elecsys p‐tau181/Aβ42 and Fujirebio Lumipulse Aβ42/Aβ40 ratios), and apolipoprotein E (*APOE*) allele testing. Amyloid PET interpretations were made by consensus visual review from experienced readers.

**Result:**

All four patients had clinical diagnoses of mild cognitive impairment or mild dementia (Table 1). Two patients had positive CSF p‐tau181/Aβ42 and Aβ42/40 ratios with negative amyloid PET (indicating sparse to no neuritic plaques). Case 1 displayed aphasia (characterized by anomia and deep dysgraphia) and imaging findings possibly compatible with semantic dementia. Case 2 displayed a gait abnormality and imaging signs of abnormal CSF dynamics. Two other patients had negative CSF p‐tau181/Aβ42 measures with positive amyloid PET (indicating moderate to frequent neuritic plaques). In those instances, the clinical syndrome and imaging studies were strongly supportive of AD and the CSF Aβ42/40 ratio was “likely positive” (Table 1). For one patient (Case 4), tau PET and Lumipulse plasma p‐tau217 concentration were both positive.

**Conclusion:**

Clinicians prescribing anti‐amyloid therapies should be aware of the possibility for CSF biomarkers to be discordant from amyloid PET status in select scenarios. These cases highlight the value of considering PET imaging in the presence of clinically atypical features, concern for mixed etiology, or strong clinical suspicion for AD despite negative CSF biomarkers. Larger series are needed to identify broader patterns contributing to biomarker discordance by assay, modality (PET versus CSF/plasma measures), and medical/neurologic comorbidities.